# Fifty Years of Natural Killer Cells: Milestones and Future Horizons

**DOI:** 10.1111/sji.70091

**Published:** 2026-01-24

**Authors:** Andreas Lundqvist, Arnika K. Wagner, Benedict J. Chambers, Carin I. M. Dahlberg, Jonas Sundbäck, Angelica Cuapio, Evren Alici, Hans‐Gustaf Ljunggren

**Affiliations:** ^1^ Department of Oncology‐Pathology Karolinska Institutet Stockholm Sweden; ^2^ Department of Medicine (Huddinge) Karolinska Institutet Stockholm Sweden; ^3^ Department of Microbiology, Tumor and Cell Biology Karolinska Institutet Stockholm Sweden

**Keywords:** immunoregulation, inhibitory receptor, missing‐self, NK cell‐based immunotherapy, NK cell education, NK cell phenotype, NK cell receptors, NK cells, tumour immunity, viral immunity

## Abstract

The discovery of natural killer (NK) cells stands among the fundamental milestones in modern immunology. To mark the 50‐year anniversary of the first publications on NK cells in 1975, a symposium was held at Karolinska Institutet in Stockholm, Sweden, on October 14, 2025. The symposium brought together scientists from across generations to reflect on the field's historical roots and future directions. The meeting traced NK cell research from its serendipitous beginnings with the identification of a previously unknown lymphocyte population capable of mediating spontaneous cytotoxicity to the conceptual proposal of ‘missing self’ recognition. Subsequent studies established the role of the first inhibitory receptor Ly49A. This was followed by the discovery of NK cell killer‐cell immunoglobulin‐like receptors (KIRs), along with a range of associated inhibitory and activating receptors, providing further insights into NK cell target recognition. The symposium then highlighted how advances in genetics, cell imaging and single‐cell technologies have revealed NK cell diversity, tissue specialisation and adaptive potential. It showcased insights from rare immunodeficiencies, tumour immunology, viral immunology and systems‐level analyses, underscoring NK cells' dual roles in cytotoxic defence and immune regulation. Increasingly, artificial intelligence (AI) is being leveraged in NK cell research. Translational developments described have bridged fundamental knowledge with clinical application, exemplified by current clinical studies of engineered NK cells, NK cell engagers and checkpoint blockade strategies. Together, these reflections underscored how five decades of NK cell research, rooted in seminal Scandinavian discoveries, have transformed from an unexpected observation into a cornerstone of immunotherapeutic potential.

## Graphical abstract

To mark 50 years of NK cell research, a 2025 Karolinska symposium traced discoveries from spontaneous cytotoxicity and “missing self” recognition to receptor biology, cellular diversity, and translational advances, highlighting NK cells’ evolution into modern immunotherapy.

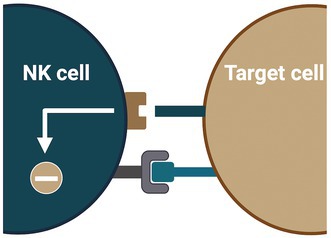

**Abbreviations**
AI: artificial intelligence
ANKET: antibody‐based NK cell engager technology
CAR: chimeric antigen receptor
CMV: cytomegalovirus
DAP: DNAX‐activating protein
GMP: good manufacturing practice
HIF: hypoxia‐inducible factor
HLA: human leukocyte antigen
HLH: hemophagocytic lymphohistiocytosis
ILC: innate lymphoid cell
iPSC: induced pluripotent stem cell
ITAM: immunoreceptor tyrosine‐based activation motif
ITIM: immunoreceptor tyrosine‐based inhibition motif
KIR: killer‐cell immunoglobulin‐like receptor
MCMV: mouse cytomegalovirus
MHC: major histocompatibility complex
MISTRG: humanised mouse strain expressing multiple human genes (M‐CSF, IL‐3/GM‐CSF, SIRPα, TPO)
NK: natural killer
NSCLC: non–small cell lung cancer
PDO: patient‐derived organoid
RUNX3: runt‐related transcription factor 3
SHP: src homology region 2 domain‐containing phosphatase
SLAM: signalling lymphocyte activation molecule
TiNK: tissue‐resident NK cell
ULBP: UL16‐binding protein

## 1 Introduction

Understanding our past is essential for guiding future scientific endeavours. In the field of immunology, reflecting on historical milestones not only honours the pioneers who paved the way but also provides invaluable insights that shape current research and drive innovation. The discovery of natural killer (NK) cells stands as a testament to such pivotal moments, illustrating how foundational research can lead to groundbreaking advancements in medical science.

In 1975, researchers at Karolinska Institutet in Stockholm, Sweden, led by Rolf Kiessling, Eva Klein and Hans Wigzell, identified NK cells, a discovery that reshaped our understanding of the immune system's role in combating cancer and viral infections [1, 2]. Over the past five decades, NK cells have evolved from being perceived as incidental immune components to becoming key players in modern immunotherapy‐related research [3, 4]. To commemorate this significant milestone, a symposium titled ‘NK Cells: 50 Years’ was held at Karolinska Institutet on October 14, 2025. The event brought together leading scientists to reflect on the historical journey of NK cell research and to explore future directions, including NK cell‐based immunotherapies and their clinical applications.

The symposium commenced with opening remarks from Hans‐Gustaf Ljunggren and Andreas Lundqvist, setting the stage for a day of insightful recollections, reflections and forward‐looking discussions by leading scientists in the field. During his remarks, Ljunggren poignantly noted that Eva Klein should have been present at the symposium, but sadly passed away earlier this year, just 3 days before reaching her 100th birthday. This acknowledgment added a deeply personal and historical resonance to the event, reminding attendees of the foundational figures whose legacies continue to shape the field.

The introductory session not only celebrated five decades of groundbreaking NK cell research but also framed the meeting as an opportunity to honour the pioneering scientists who shaped the field, revisit transformative discoveries and explore how current advances continue to redefine our understanding of innate immunity and its therapeutic potential.

## 2 The Discovery and Naming of Natural Killer (NK) Cells

The discovery of NK cells is a remarkable example of serendipity in modern immunology. It took place at Karolinska Institutet in Stockholm in the early 1970s, within the intellectually vibrant environment created by Eva and Georg Klein at the Department of Tumour Biology. Their laboratory attracted an international community of young scientists who explored tumour immunology and viral oncogenesis at a time when the immune system was largely viewed through the lens of adaptive T‐ and B‐cell responses.

Among these young researchers was Rolf Kiessling, a PhD student supervised by Eva Klein and Hans Wigzell. His thesis aimed to define cytotoxic T cell responses against Moloney virus–induced lymphomas, using the murine tumour line YAC‐1 as a model target. Kiessling described how he tested spleen cells from both immunised and non‐immunised mice in the newly developed chromium‐release assay and noticed an unexpected and frustrating phenomenon: even cells from completely normal, unprimed animals efficiently killed YAC‐1 tumour cell targets.

**FIGURE 1:**
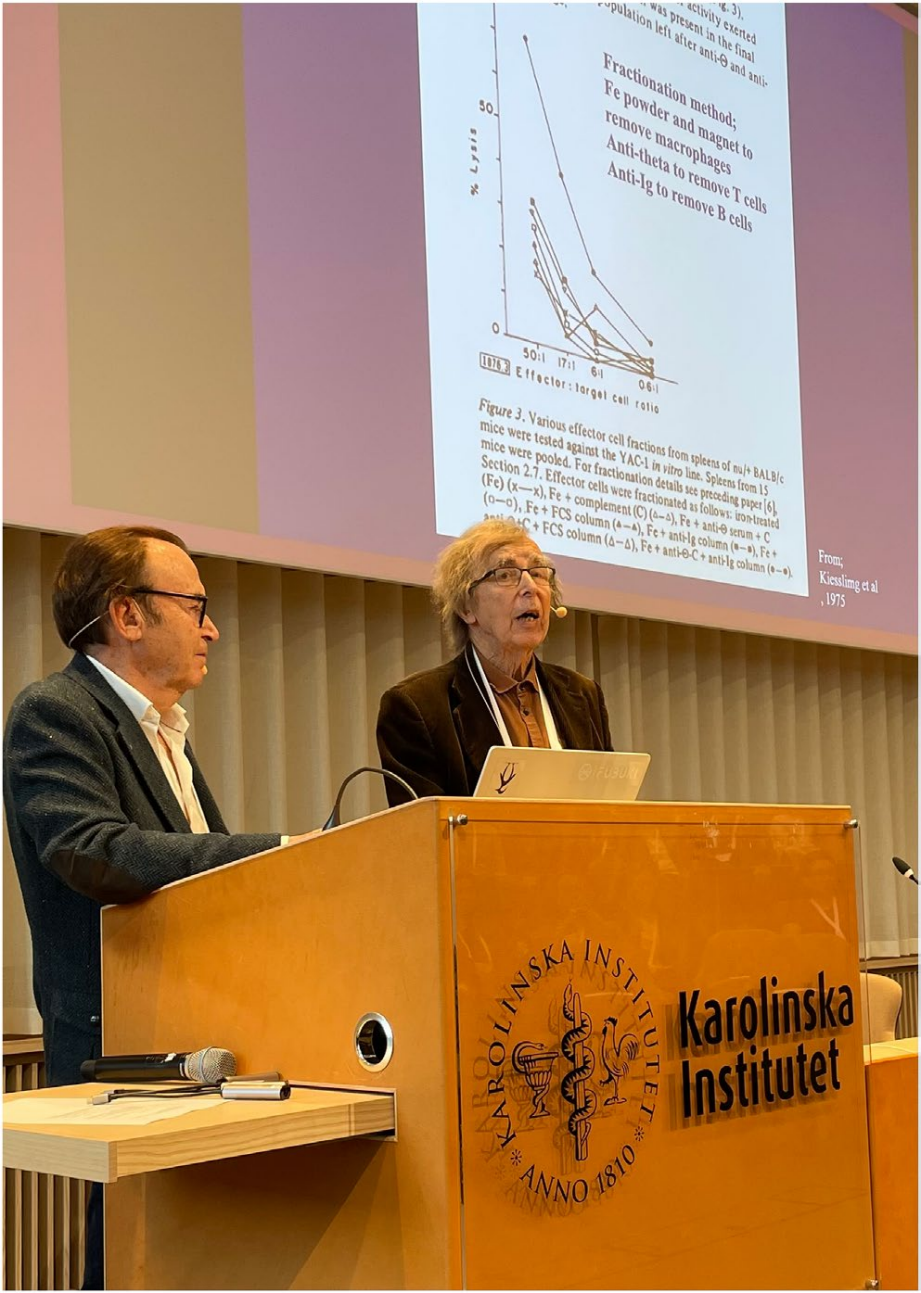
Rolf Kiessling and Hans Wigzell revisiting the discovery of NK cells. At the symposium ‘Fifty Years of Natural Killer Cells: From Discovery to Therapeutic Frontiers’, held at Karolinska Institutet in Stockholm, Sweden, the opening lecture was jointly delivered by Rolf Kiessling (left) and Hans Wigzell (right). Displayed on the screen is an original figure from one of their two seminal 1975 back‐to‐back publications describing the discovery and naming of natural killer (NK) cells. The figure shows that spleen cells retained and, in some cases, increased spontaneous cytotoxicity against murine YAC‐1 lymphoma cells even after depletion of macrophages, T cells and B cells. These and other related observations provided compelling evidence for a distinct lymphocyte population responsible for spontaneous cytotoxicity, which Kiessling, Wigzell and co‐author Eva Klein termed NK cells.

What initially appeared as annoying ‘background noise’ soon became the main project. Through a series of meticulous depletion experiments, removing macrophages, T cells and B cells, Kiessling and colleagues demonstrated that the cytotoxicity remained, or even increased, when known lymphocyte subsets were eliminated (Figure 1). The data pointed to a new effector cell population that was neither T nor B cell, but nevertheless displayed potent, spontaneous killing of certain tumour and virus‐infected cells. In 1975, these observations were published in the *European Journal of Immunology*, marking the formal discovery of a new, previously undefined cytotoxic cell [1, 2]. At roughly the same time, Ronald B. Herberman and coworkers in the United States independently reported ‘natural cytotoxic reactivity’ of lymphoid cells against tumours [5, 6]. Together, these parallel findings established the existence of a constitutive antitumor effector mechanism within the normal immune system.

The new cell type still lacked a proper name. During the preparation of the first manuscripts, Kiessling proposed the term ‘NKL cells’, for ‘natural killer of lymphoma cells’, by analogy to ‘*natural antibodies*’ [1, 2]. In the corridors of the Tumour Biology Department, Eva Klein wisely suggested simplifying it to natural killer (NK) cells, emphasising their innate and general nature rather than a tumour‐restricted specificity. The term quickly gained acceptance and endures to this day.

Hans Wigzell and Rolf Kiessling reminded everyone that the serendipitous discovery of NK cells is beautifully captured in the famous quote by Isaac Asimov: ‘The most exciting phrase to hear in science, the one that heralds discoveries, is not “Eureka!” (I found it!) but “That's funny…”’. This reflection underscored the unexpected nature of scientific breakthroughs and the importance of curiosity and observation in driving discovery, an idea that resonates deeply with the origins of NK cell research.

The discovery of NK cells profoundly changed the conceptual framework of immunology. It revealed that the immune system possessed not only adaptive, antigen‐specific lymphocytes, but also innate lymphocytes capable of recognising abnormal cells through other cues. In the 1980s, Klas Kärre, another student from the Klein laboratory supervised by Kiessling, articulated the ‘missing self’ hypothesis while writing up his research as a doctoral thesis (presented in 1981), proposing that NK cells have the capacity to detect the ‘absence of normal self‐MHC class I molecules’ on target cells.

## 3 Target Cell Recognition and the Missing Self‐Hypothesis

At the meeting, Kärre offered a historical and conceptual reflection on the origins of the ‘missing self’ hypothesis, an insight that arose during the preparation of his PhD thesis and went on to transform how immunologists today understand NK cell recognition. Drawing an analogy to Sweden's attempts in the early 1980s to distinguish foreign from domestic submarines [7, 8], Kärre likened the immune system's challenge to that of recognising ‘self’ versus ‘non‐self’ with strategies adopted by the Swedish Navy at the time. The key insight was that NK cells might function by inactivation upon self‐recognition rather than by activation upon detecting foreign antigens; thus, cells missing ‘self’ signals (such as MHC class I molecules) would be targeted for destruction.

Klas illustrated this concept by recounting his exploration of the hypothesis through the studies of *Botryllus schlosseri* , a colonial tunicate that forms vascular fusions only with genetically compatible individuals through an allorecognition system mediated by highly polymorphic histocompatibility genes [9]. He further drew a parallel to turkey hens, which use a two‐signal system to identify their chicks: first by visual cues and then by auditory cues, described by Wolfgang Schleidt in his book *Signale der Tierwelt* [10]. These analogies helped clarify how NK cells might integrate multiple signals to determine whether a cell should be eliminated or not.

Kärre, in part, traced the intellectual development of the idea from studies of hybrid resistance in mice, where F_1_‐hybrids rejected parental bone marrow grafts [11]. Other early studies indicated that tumour cells with reduced MHC class I expression were NK‐sensitive [12, 13], whereas interferon treatment, which increased MHC I, rendered them resistant [14, 15]. Additional confirmatory studies further substantiated the notion of NK cell sensitivity of MHC class I‐deficient cells [16, 17, 18, 19, 20, 21]. Taken together, the studies suggested that MHC class I molecules transmit inhibitory signals to NK cells, preventing attack on normal, healthy cells. Kärre recounted the iterative reasoning process leading to his ideas and conclusion, including the conceptual shift from describing what NK cells *kill* to what they *do not kill* [4].

**FIGURE 2:**
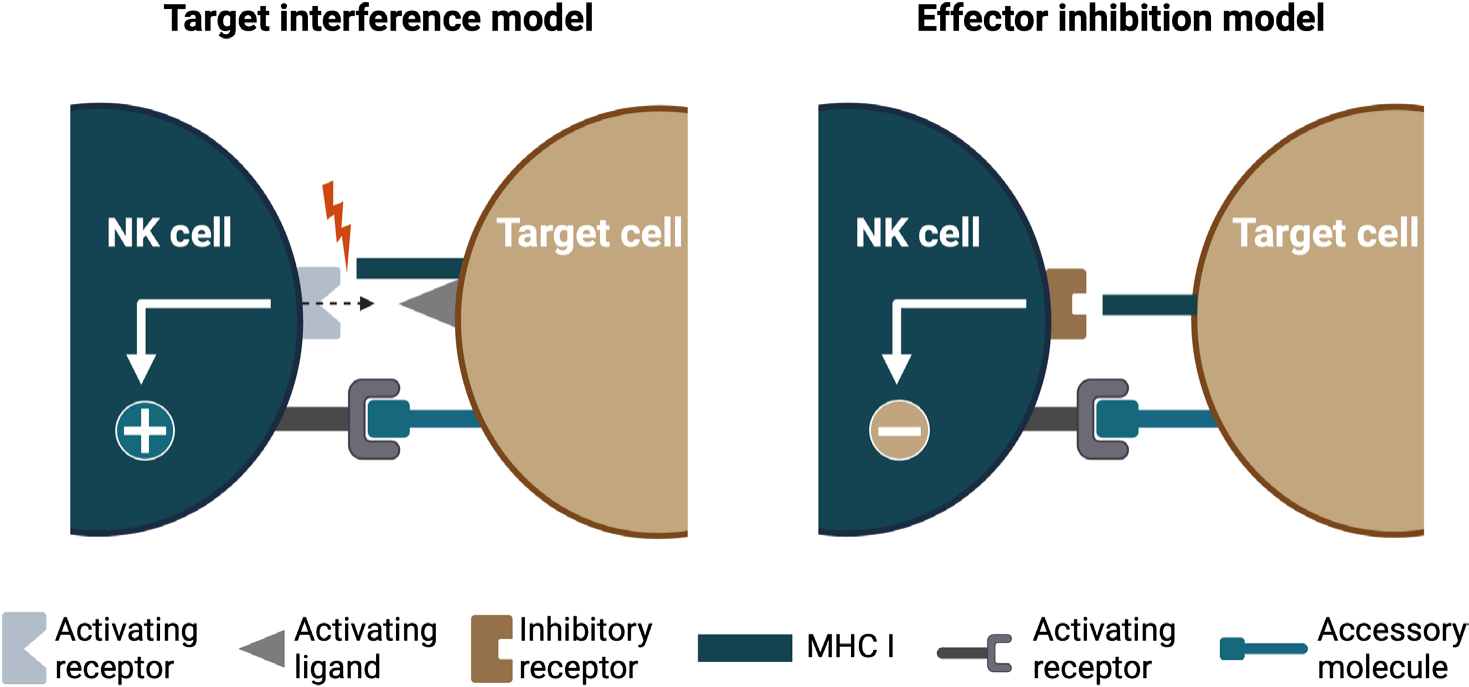
Early models of ‘missing self’ recognition. Illustration of early conceptual models explaining how target cell MHC class I expression could inhibit NK cell‐mediated cytotoxicity [22]. Both displayed models sought to explain the observation that NK cell activation could be suppressed by a target cell‐dependent, MHC class I‐mediated event. (Left) Target interference model: An ‘accessory molecule’ was hypothesized to be recognised by an activating NK cell receptor. Its surface expression was proposed to be either prevented or sterically hindered by MHC class I molecules. (Right) Effector inhibition model: MHC class I molecules were proposed to be directly recognised by an inhibitory NK cell receptor. This model required the presence of ‘accessory molecules’ to initiate binding and potentially activate NK cell effector function [22]. Later studies, including the initial identification of NK cell inhibitory receptors that bind MHC class I, provided definite support for the ‘effector inhibition’ model. Subsequent discovery of NK cell activating receptors further refined this conceptual framework. Noteworthy, the cited 1990 *Immunology Today* paper also contains the first published use of the term ‘*missing self*’ in context of NK cell recognition of MHC class I‐deficient target cells. Figure created with BioRender.com.

Kärre, in his talk, also reflected on broader principles of scientific discovery. He discussed the balance between intellectual simplicity, empirical consistency and testability. He acknowledged that early versions of the model failed to account for all data, some of which were later shown to be caused by, for example, variable expression of activating ligands, but emphasised that its core concept survived because of its logic and predictive power (Figure 2). Finally, Kärre credited the collaborative and critical climate within the NK field. ‘Missing self’, he concluded, illustrates how an elegant biological principle can arise from reconciling paradoxes and redefining the boundaries of self‐recognition.

## 4 Towards the Identification of NK Cell Receptors

Wayne Yokoyama then provided a recollection of research behind the first direct molecular demonstration that NK cells can specifically recognise MHC class I molecules through inhibitory receptors, offering further mechanistic insights to Kärre's ‘missing self’ hypothesis. Using IL‐2–activated NK cells from C57BL/6 mice, he and colleagues separated cells based on expression of the Ly‐49A receptor, newly discovered by his group. He found Ly‐49A^+^ NK cells to be unable to lyse tumour targets bearing H‐2^d^ or H‐2^k^ MHC class I alleles, whereas Ly‐49^−^ NK cells killed them efficiently [23]. To dissect this specificity, Yokoyama and collaborators transfected the MHC class I gene *H‐2D* ^*d*^ into susceptible C1498 (H‐2^b^) tumour cells. Expression of H‐2D^d^ rendered these targets resistant to Ly‐49^+^ NK cells but not to Ly‐49^−^ cells, demonstrating that H‐2D^d^ conferred protection. Importantly, this resistance was reversed by monoclonal antibodies against Ly‐49A or the α1/α2 domains of H‐2D^d^, confirming a direct interaction between Ly‐49 and the peptide‐binding region of MHC class I molecules.

These experiments established Ly‐49A as an inhibitory NK receptor and demonstrated that NK cells possess receptors capable of specific engagement with self‐MHC molecules, transmitting signals that prevent killing [24]. The study suggested that NK cells are equipped with inhibitory receptors that monitor the expression of self‐MHC on potential targets; if MHC is missing or altered, inhibition is lost and cytotoxicity is triggered. The work also proposed that Ly‐49A belongs to a multigene family within the NK gene complex, hinting at the diversity of MHC‐recognising NK receptors later confirmed in both mice (Ly49 family) and humans (KIRs) [25].

These studies transformed the understanding of NK cell specificity, providing the molecular foundation for the missing‐self model and the start of a new era of research into inhibitory and activating receptor signalling, NK tolerance and self‐recognition across species, as very nicely described by Yokoyama.

## 5 Memorable Events in the Years That Followed the NK Cell Discovery and Early Work

From the early discoveries in the 1970s, 1980s and early 1990s, the past three decades have transformed NK cell biology from a descriptive field into a molecular and translational discipline, as comprehensively reviewed by Lewis Lanier [3].

By the early 1990s, the *missing‐self* hypothesis, proposing that NK cells kill targets lacking MHC class I, predicted inhibitory receptors for self‐MHC. These were soon identified as the mouse Ly49 family (as described above) and human KIRs, defining the first ‘immune checkpoints’. Studies showed that NK cell activation depends on a balance between inhibitory MHC‐recognising receptors and activating receptors.

In his talk, Lanier described how activating ‘natural cytotoxicity receptors’, NKp30, NKp44 and NKp46 were cloned in the late 1990s, and key signalling adaptors were defined [26]. The ITAM‐bearing DAP12 and DAP10 molecules were found to associate with activating receptors, while inhibitory receptors contained ITIM motifs recruiting SHP‐1/2 phosphatases [27, 28]. The stress‐induced NKG2D receptor and its ligands (MICA/B, ULBPs in humans; Rae1, H60, Mult1 in mice) established the principle of ‘altered‐self’ recognition. Clinical observations linked donor–recipient KIR mismatches in haematopoietic transplantation to reduced leukaemia relapse, foreshadowing NK‐based immunotherapy [29].

Around the year 2000, direct viral recognition by NK cells was demonstrated when mouse cytomegalovirus (MCMV) m157 was identified as a ligand for the activating Ly49H receptor, an evolutionary arms race example showing paired activating and inhibitory receptors [30]. Similar mechanisms were later found for human CMV with CD94/NKG2C [31]. A major conceptual shift came with the recognition that NK cells can form immunological memory. Mouse Ly49H^+^ NK cells undergo clonal expansion and persist after murine (MCMV) infection, providing antigen‐specific recall responses [32]. Human CMV infection analogously expands long‐lived NKG2C^+^ NK cells [31, 33].

At the same time, the concept of NK cell education or licensing emerged; NK cells bearing self‐MHC‐specific inhibitory receptors become functionally competent, whereas those lacking them are hyporesponsive [34]. This reframed inhibitory signalling as a requirement for proper NK activation thresholds.

High‐dimensional analyses revolutionised the understanding of NK heterogeneity. Mass cytometry (CyTOF) revealed 10s of 1000s of phenotypic NK subsets in healthy humans, largely due to variegated KIR expression [35]. Single‐cell RNA sequencing confirmed profound transcriptional diversity [36]. Epigenetic profiling demonstrated that CMV infection imprints stable, heritable changes on NK cells [37]. Human ‘adaptive’ NK cells silence *FCER1G* and *SYK*, rely on *CD3ζ* and *ZAP70* for signalling, and exhibit enhanced antibody‐dependent cytotoxicity, mirroring adaptive lymphocytes [38]. In mice, Ly49H^+^ memory NK cells display distinct chromatin landscapes but retain classical NK signalling components [39].

Modern gene‐editing and transduction technologies have propelled NK cells into clinical oncology. CAR‐engineered NK cells, first tested against CD19^+^ malignancies in 2020, showed safety and efficacy without graft‐versus‐host disease [40]. Ongoing efforts aim to extend NK cell persistence, overcome suppression in solid tumours and develop NK cell engagers, multimeric antibodies linking activating NK receptors to tumour antigens.

Parallel advances highlight NK cells' regulatory roles: production of IFN‐γ bridges innate and adaptive immunity, while secretion of IL‐10 can restrain excessive T‐cell responses or tissue pathology. NK–dendritic cell crosstalk via cytokines such as IL‐12, IL‐15 and IL‐18 underpins early antiviral and antitumor defences. Tissue‐resident and decidual NK cells exemplify specialised local adaptations [41, 42], and the broader family of innate lymphoid cells (ILCs) has been delineated from classical cytotoxic NK lineages [43].

Hence, as concluded by Lanier, from the 1990s onward, NK biology has evolved from identifying inhibitory checkpoints to engineering therapeutic effectors. Today's questions concern the molecular basis of NK memory, mechanisms of tolerance and exhaustion, and strategies to re‐energise NK cells within tumours or exploit their regulatory potential in autoimmunity. Fifty years after their discovery, NK cells stand at the intersection of basic immunology and translational medicine, poised for another era of unexpected insights.

## 6 From Historical Recollections to Today's NK Cell Research Front‐Line

Building on five decades of discovery, from the initial recognition of ‘natural’ cytotoxicity to the molecular dissection of activating and inhibitory receptors, NK cell biology has matured into a multidimensional field spanning genetics, signalling, tissue adaptation, super‐resolution microscopy, multi‐omics analyses and therapy. The classical questions of ‘how NK cells recognize targets’ and ‘what defines an NK cell’ have evolved into precision analyses of lineage specification, education and plasticity in health and disease. Enabled by single‐cell technologies, patient genomics and engineered models, current research now links developmental checkpoints to immune regulation and clinical translation. The following examples illustrate this dynamic frontier, where insights from rare immune disorders, tissue immunology and translational innovation converge to redefine NK cell identity and therapeutic potential.

Emily Mace presented advances in defining human NK cell development and effector specialisation, emphasising how cellular architecture and tissue context shape cytotoxic function. Her group employs high‐resolution imaging and single cell approaches to link cytoskeletal dynamics and vesicle polarisation to immune synapse efficiency and maturation [44]. Recent work on rare immunodeficiencies revealed that monogenic variants in CMG (Cdc45‐MCM‐GINS) replisome proteins, including GINS4, can cause human NK cell deficiency with variable expressivity [45, 46]. In affected siblings, cell‐cycle impairment and apoptosis arose after NK lineage commitment, and allelic bias during differentiation correlated with disease severity, identifying a new mechanism by which differential allelic expression modulates monogenic disease outcomes. Together, these studies integrate molecular cell biology with human genetics to explain how NK cells acquire structure and function, and how developmental and transcriptional checkpoints govern their resilience across tissues and disease states.

Yenan Bryceson first highlighted the importance of synergies among receptors on NK cells for the activation of cytotoxicity and cytokine release [47, 48, 49]. Then he described how studies of rare immune deficiencies have illuminated the molecular basis of NK cell cytotoxicity and set today's diagnostic standards. His group pioneered the use of the CD107a degranulation assay in primary immunodeficiency related studies, now being routine in evaluating exocytic defects and hemophagocytic lymphohistiocytosis (HLH). Through combined genetic and functional studies, his group has mapped pathways that govern cytotoxic granule release, identifying key roles for Munc13‐4, syntaxin‐11 and other effectors [50, 51, 52]. More recently, Bryceson's work has refined concepts of receptor synergy and SLAM‐family signalling via the EAT‐2 adaptor, explaining how these networks tune killing in resting cells and define NK functional identity across adaptive‐like states [53]. Together, these insights link fundamental signalling architecture to clinical phenotypes and continue to inform the classification and management of inherited cytotoxicity disorders.

Björn Önfelt showcased pioneering work combining quantitative imaging and single cell analyses to reveal the heterogeneity of NK cell killing. Using microchip‐based cytotoxicity assays and live‐cell microscopy, his group demonstrated that a small fraction of ‘serial killer’ NK cells accounts for the majority of target cell elimination [54, 55]. By correlating dynamic imaging with transcriptomic and phenotypic data, they have identified predictive features of these high‐performing cells and proposed models of NK efficiency governed by immune synapse formation and recycling kinetics [54, 56]. Recent studies extend these principles to interactions with tumour spheroids and infected cells, showing that tissue context and target resistance modulate serial killing capacity [57, 58]. These and other AI‐based findings reshape our understanding of NK functional diversity, providing both mechanistic insight and quantitative frameworks relevant for optimising NK‐based immunotherapies.

Nicole Marquardt summarised advances in defining tissue and tumour–resident NK cell programs, particularly within the human lung. Integrating pan‐tissue single‐cell datasets and in‐depth profiling of non–small cell lung cancer (NSCLC), her group identified distinct CD49a^+^CD16^+^ NK subsets with tissue‐specific transcriptional signatures and hypoxia‐responsive modules [59, 60, 61]. These intratumoral NK populations exhibit limited perforin expression and low classical checkpoint levels, indicating a unique balance between residency and effector restraint. Building on human lung tissue‐resident NK (TiNK) datasets, ongoing work dissects the differentiation trajectories of these subsets and their contribution to tumour control. By linking adaptive‐like CD56^bright^ tissue‐resident cells to clinical outcomes, Marquardt's studies provide a blueprint for understanding site‐specific NK adaptation and for developing strategies to restore NK cytotoxicity in solid tumours.

Adelheid Cerwenka revisited the NKG2D–ligand axis, tracing its discovery from the mouse ligand Rae‐1 to its roles in human cancer [62]. Her group has systematically explored how NKG2D ligand expression and class I HLA status determine susceptibility of tumour organoids, including colorectal patient‐derived organoids (PDOs), to NK cell attack. Current work broadens this framework to include non‐classical inhibitory checkpoints and the metabolic and hypoxic cues that limit NK function in tumours [63]. In particular, she demonstrated that targeting hypoxia‐inducible factors (such as HIF‐2α) can reinvigorate NK cytotoxicity against PDOs [63, 64]. This integrative approach, from receptor‐ligand biology to tumour immunometabolism, clarifies how NK activation thresholds are shaped within the tumour microenvironment and guides rational design of interventions to restore NKG2D‐dependent antitumor responses.

Annika Niehrs proposed a dynamic model of NK cell tissue residency and egress centered on the CD56^bright^ subset [65]. Through comparative profiling of human tissues, lymph and transplant biopsies, she identified CD69^high^ NK populations with distinct residency markers and site‐specific transcriptional programs. Analyses of lymph fluid and lymph‐node compartments revealed specialised lymph‐resident subsets, suggesting bidirectional trafficking between lymphoid and peripheral tissues. Using humanised MISTRG mice, her group demonstrated preferential extravasation of CD56^bright^ NK cells and defined RUNX3 as a key transcriptional regulator of tissue residency [65]. Together, these studies support a revised view of NK cell compartmentalization, emphasising the plasticity and circulation routes of human tissue‐resident NK cells and their potential for therapeutic modulation.

Aline Pfefferle presented comprehensive single‐cell atlases capturing NK differentiation from CD56^bright^ to CD56^dim^ states, integrating blood, tissue and tumour contexts [66]. Meta‐analyses conducted across multiple cancer types revealed conserved transcriptional programs imposed by the tumour microenvironment, characterised by effector dampening, exhaustion‐like modules and selective loss of cytotoxic genes. Despite this convergence, lineage‐imprinted features remain detectable, underscoring intrinsic diversity within infiltrating NK populations [66]. These datasets form the foundation for identifying therapeutic targets aimed at ‘unlocking’ suppressed cytotoxic potential after tumour infiltration. By distinguishing lineage trajectories from tumour‐imposed signatures, Pfefferle's work provides a high‐resolution framework for rational engineering and reprogramming of NK cells for cancer immunotherapy [67].

Eric Vivier connected foundational NK cell biology to translational immunotherapy. His group delineated three principal circulating NK subsets (NK1/NK2/NK3) with distinct receptor repertoires and effector profiles, correlating these to distributions across tumour microenvironments [36]. Clinically, Vivier's work underpins the development of *monalizumab*, an anti‐NKG2A antibody now evaluated in lung cancer and other malignancies through collaborations with industry [68]. Beyond checkpoint blockade, he introduced the ANKET platform, tri‐ and tetra‐specific NK cell engagers that combine NKp46/CD16 triggering with tumour antigen targeting and IL‐2 variant delivery to potentiate selective NK activation [69, 70]. These efforts exemplify how mechanistic NK receptor biology translates into drug development, bridging academic discovery and biopharmaceutical innovation.

Karl‐Johan Malmberg finally focused his presentation on NK cell education through KIR–HLA interactions and its lasting imprint on cytotoxic granule content and function. His group linked the strength of NK education to remodelling of secretory lysosomes, thereby explaining functional variability among licensed cells [71]. Building on this foundation, his translational program develops adaptive NK cell–based therapies derived from super donors with potent missing‐self reactivity [72]. GMP‐grade single‐KIR^+^ NKG2C^+^ adaptive NK products show strong persistence and cytotoxic potential and efforts are underway to integrate additional modules, such as NKG2A/C checkpoint switches and iPSC‐based derivation routes. These studies bridge fundamental NK education biology with clinical translation, shaping the next generation of targeted NK cell immunotherapies [67].

**FIGURE 3:**
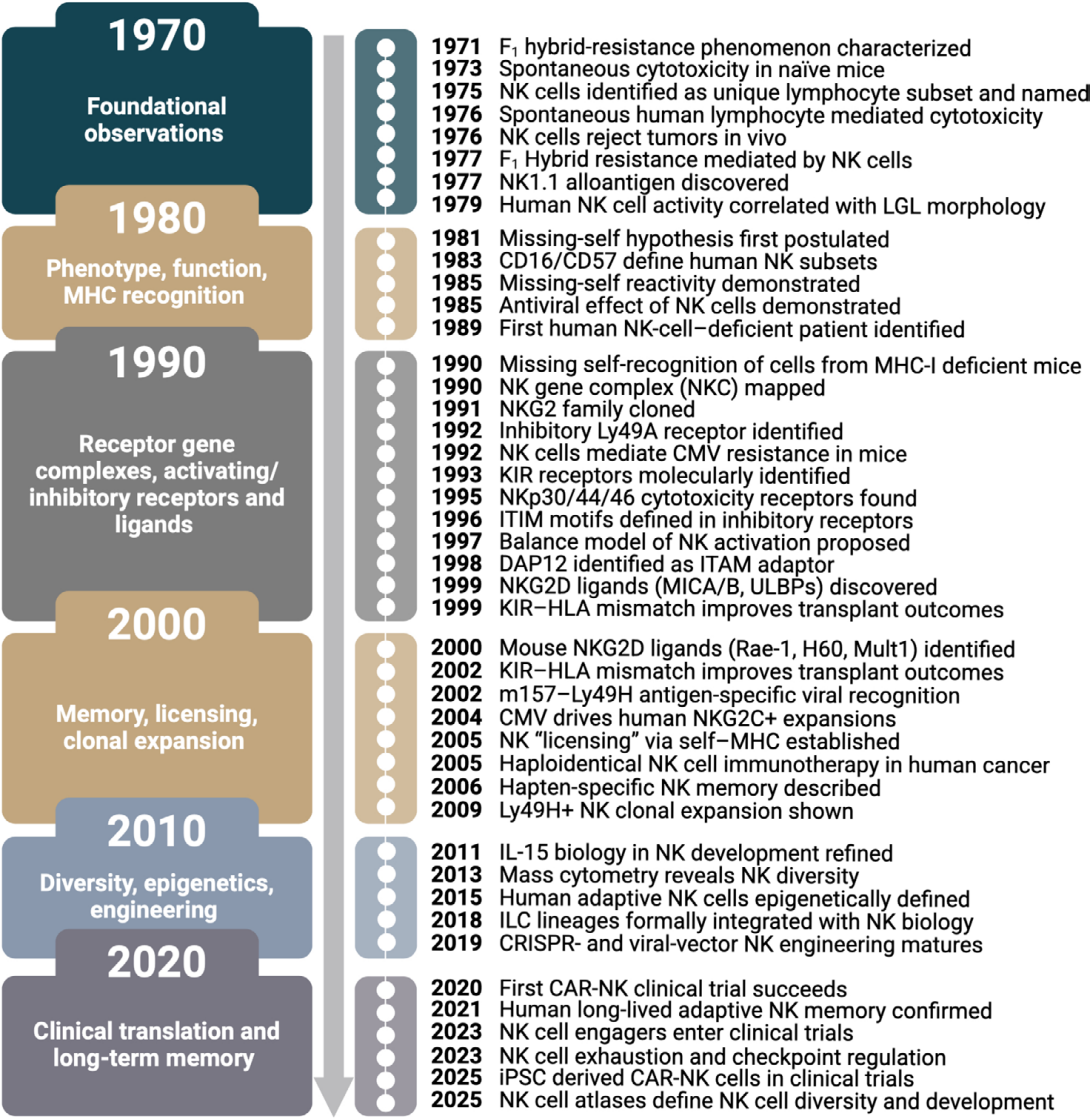
Fifty years of key discoveries in NK cell biology. Chronological overview of seminal discoveries that have shaped the field of NK cell biology from 1970 to 2025. The selected milestones highlight only a few of the many important contributions that have advanced our understanding of NK cell identity, phenotype, function and broader roles in immunity and immunotherapy. Figure created with BioRender.com.

Collectively, these talks highlighted the unique culture that has shaped NK cell research. Over the years, the NK cell community has been remarkably open and collaborative, a spirit that has greatly contributed to the field's rapid progress and enduring impact. This symposium served not only as a retrospective of the last 50 years of NK cell research but also as a reminder of the importance of reflecting on our scientific heritage (Figure 3).

## 7 Conclusion

**FIGURE 4:**
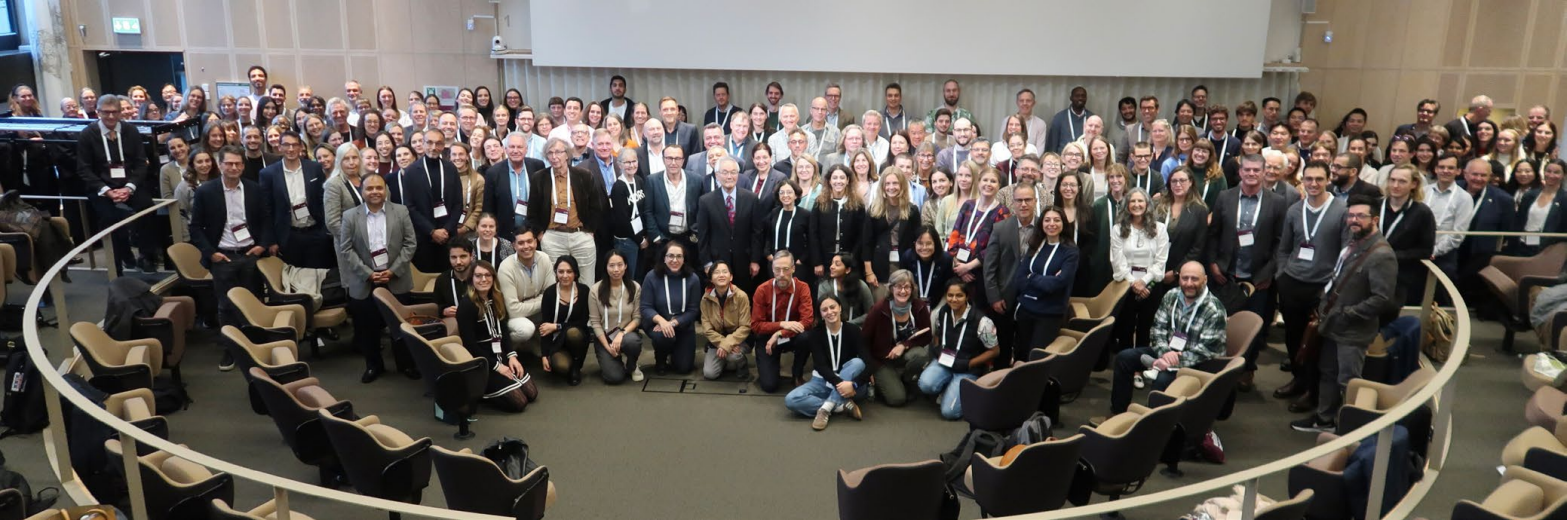
Participants of the NK Cell 50th Anniversary Symposium at Karolinska Institutet. Group photo of attendees at the symposium ‘Fifty Years of Natural Killer Cells: From Discovery to Therapeutic Frontiers’, held at Karolinska Institutet, Stockholm, Sweden, on October 14, 2025. The meeting celebrated the 50th anniversary of the first scientific publications describing natural killer (NK) cells in 1975 and brought together researchers across generations to discuss the history, scientific progress and future directions of NK cell biology.

Revisiting the foundational discoveries of NK cells at the present meeting allowed students and established scientists in the field to appreciate the evolution of ideas, experimental approaches and conceptual frameworks that have led to current advancements. Such retrospection not only honoured the contributions of pioneering scientists but also provides context for ongoing research and inspires future innovations. In the rapidly evolving field of immunology, where new therapies and technologies emerge continually, grounding our understanding in a historical context ensures that progress remains both informed and purposeful. The journey of NK cells, from their initial discovery to their current role as powerful therapeutic agents, illustrates how past insights illuminate the path forward. As researchers continue to push the boundaries of NK cell‐based therapies, the lessons from five decades of research serve as guiding principles, shaping a future where NK cells can be fully harnessed to improve human health. As we celebrate these milestones, we are reminded that the continuum of scientific discovery is built upon the foundations laid by previous generations. Embracing this perspective fosters a culture of appreciation, curiosity and intellectual humility, driving the relentless pursuit of knowledge that propels science and medicine forward (Figure 4).

## Funding

This work was supported by the Swedish Cancer Society, Sweden's Innovation Agency, Wenner‐Gren Foundations, the Cancer Research Funds of Radiumhemmet and the Scandinavian Foundation for Immunology and Karolinska Institutet.

## Ethics Statement

The authors have nothing to report.

## Conflicts of Interest

All authors have been active contributors to the field of NK cell research, including studies discussed in this meeting report. A.K.W., C.I.M.D., E.A. and H.‐G.L. have participated in corporate collaborations and activities related to the development of NK cell–based immunotherapies. H.‐G.L. also serves on the editorial board of the *Scandinavian Journal of Immunology*.

## Data Availability

The data that support the findings of this study are available on request from the corresponding author. The data are not publicly available due to privacy or ethical restrictions.
